# Quadratic Spin–Orbit Mechanism of the Electronic g-Tensor

**DOI:** 10.1021/acs.jctc.2c01213

**Published:** 2023-03-10

**Authors:** Petra Pikulová, Debora Misenkova, Radek Marek, Stanislav Komorovsky, Jan Novotný

**Affiliations:** † CEITEC—Central European Institute of Technology, Masaryk University, Kamenice 5, Brno CZ-62500, Czechia; ‡ Department of Chemistry, Faculty of Science, Masaryk University, Kamenice 5, Brno CZ-62500, Czechia; § Institute of Inorganic Chemistry, Slovak Academy of Science, Dúbravská cesta 9, Bratislava SK-84536, Slovakia

## Abstract

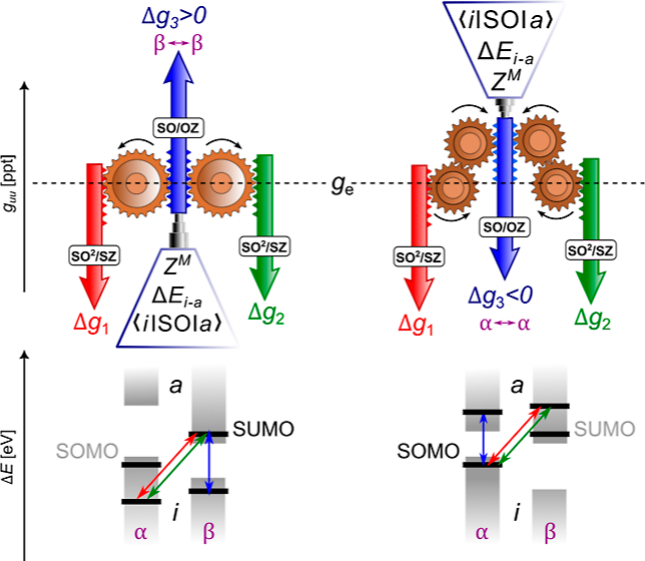

Understanding how the electronic g-tensor is linked to the electronic structure is desirable for the correct interpretation of electron paramagnetic resonance spectra. For heavy-element compounds with large spin–orbit (SO) effects, this is still not completely clear. We report our investigation of quadratic SO contributions to the g-shift in heavy transition metal complexes. We implemented third-order perturbation theory in order to analyze the contributions arising from frontier molecular spin orbitals (MSOs). We show that the dominant quadratic SO term—spin-Zeeman (SO^2^/SZ)—generally makes a negative contribution to the g-shift, irrespective of the particular electronic configuration or molecular symmetry. We further analyze how the SO^2^/SZ contribution adds to or subtracts from the linear orbital-Zeeman (SO/OZ) contribution to the individual principal components of the g-tensor. Our study suggests that the SO^2^/SZ mechanism decreases the anisotropy of the g-tensor in early transition metal complexes and increases it in late transition metal complexes. Finally, we apply MSO analysis to the investigation of g-tensor trends in a set of closely related Ir and Rh pincer complexes and evaluate the influence of different chemical factors (the nuclear charge of the central atom and the terminal ligand) on the magnitudes of the g-shifts. We expect our conclusions to aid the understanding of spectra in magnetic resonance investigations of heavy transition metal compounds.

## 1 Introduction

The techniques of magnetic resonance spectroscopy represent indispensable tools in the arsenal of modern chemists, physicists, and biologists. The central parameters of nuclear magnetic resonance (NMR) and electron paramagnetic resonance (EPR) are the chemical shift tensor (δ) and the electronic g-tensor (**g**), respectively. They characterize the effects of the surrounding electrons and induced electronic currents on the resonance frequency of the probing nucleus (NMR) or electron (EPR). The resonance characteristics are well known to be greatly affected by relativistic effects in molecules containing atom(s) of heavy element(s).^1−3^

The g-tensor contains invaluable information about the molecular geometry and electronic structure of paramagnetic substances. The g-tensor is part of the EPR effective spin Hamiltonian that describes the effect of an external uniform magnetic field (in a linear regime) on the electronic structure. From the mathematical point of view, the purpose of this effective Hamiltonian is to parametrize the projection of the quantum mechanical Hamiltonian onto the subspace formed by the populated eigenstates. The g-tensor can then be viewed as a set of parameters that parametrize the magnetic moments of the electronic states from this subspace. At the same time, these electronic states are represented in the effective (fictitious) spin space by the (half-) integer spin. Therefore, the eigenvalues of the g-tensor play a role as effective g-factors of a molecule that are, in the general case, different for each principal orientation.^4−6^

The deviation of the molecular g-factors from the g-factor of an ideal spin is caused mainly by the spin–orbit coupling (SOC) interaction. Although linear spin–orbit (SO) effects dominate many properties, higher than linear contributions are responsible for the SO effects on the electron density^7,8^ and interatomic distances^9^ and have been demonstrated to affect also the NMR shift tensor,^10^ indirect spin–spin coupling tensor,^11^ electronic g-tensor,^12−15^ and hyperfine A-tensor.^15^ For systems containing only light elements, inclusion of only the linear SOC effects is usually sufficient.^16^ However, in cases of small highest occupied molecular orbital–lowest unoccupied molecular orbital gaps, one should consider including higher-order SOC effects even for relatively light systems (see, for example, the strong quadratic SOC contribution in the SeO molecule^17^). The higher than linear SOC effects should not be underestimated when an atom of a heavy element is present in the studied system.^14^ In the framework of density functional theory (DFT), the use of methods that include SOC interaction variationally (self-consistently) is recommended,^17−21^ while within multi-reference wavefunction methods, one should employ a one- or two-step procedure to include SOC, as is described in refs (6), (22)–^26^. Currently, the state-of-the-art DFT approach for predicting the g-tensor is based on the Dirac–Coulomb Hamiltonian, utilizes noncollinear Kramers unrestricted DFT methodology, and uses either the restricted kinetically balanced basis^18^ or the restricted magnetically balanced basis and London atomic orbitals.^21^

The structure of low-spin 4d^7^ and 5d^7^ PNP pincer complexes is well-suited for the investigation of the electronic origin of the g-shift in a DFT framework thanks to (i) a doublet electron configuration, (ii) the presence of one dominant magnetic coupling between molecular orbitals, which gives rise to large g-shifts (including large quadratic contributions, as we demonstrate below), and (iii) the availability of experimental data for a series of diversely substituted compounds. In our previous theoretical work on Ir pincer complexes,^15^ we demonstrated that second-order perturbation theory (PT)—including only linear SO effects—provides a transparent analysis complementary to the variational treatment as it allows qualitative interpretation of the molecular spin orbital (MSO) contributions to the g-tensor in the language of chemists. However, substantial higher than linear SO contributions to the g-tensor were evident in the Ir(II) (5d^7^) and Ir(IV) (5d^5^) complexes investigated, which limited the usefulness of the analysis. In particular, whereas the sign of the isotropic g-shift was reproduced correctly by accounting only for linear effects, the results for the individual g-shift components were mixed. This is related to the long-known failure^27^ of second-order PT to produce the negative Δ*g*_∥_ in linear diatomic radicals, a problem that is best avoided using variational methods. It has been shown that the negative Δ*g*_∥_ can be attributed to the quadratic contribution to the spin-Zeeman (SZ) g-shift.^28^ The insight gained from second-order PT in our previous paper and the hope that further understanding might be only one order in the perturbation away motivated us to implement the necessary tools and extend our PT-based analysis of the MSO contributions to the g-tensor to terms quadratic in SO coupling.

## 2 Theoretical Background

The four-component (4c) calculations presented in this work have been performed with the noncollinear Kramers unrestricted Dirac–Kohn–Sham (DKS) approach.^18^ This methodology is based on the Dirac–Coulomb Hamiltonian: it therefore includes one-electron (nuclear) spin–orbit and two-electron spin–same-orbit interactions, while it omits spin–other-orbit contributions. In general, 4c methods take relativistic effects into account variationally (self-consistently), *i.e.*, all orders of scalar and spin–orbit effects are considered, in contrast to PT-based methods. The 4c method utilizes a common gauge origin, which allows a more transparent analysis of individual contributions to the g-tensor (compared to methods that are based on London atomic orbitals) and is thus suitable for the purposes of this work. In the Supporting Information, we provide the decomposition of the four-component working equations presented in ref (18) to spin-Zeeman (SZ), orbital-Zeeman (OZ), and the remaining relativistic contributions
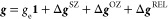
1

Here, the relativistic component Δ***g***^REL^ is usually much smaller than the SZ and OZ contributions, and we will therefore omit it in the forthcoming analysis. In the following, we will refer to results obtained by eq 1 as four-component (4c) results.

The theoretical foundations for perturbational relativistic treatment of the g-tensor with contributions up to  [in contrast to the usual ] were established in refs (28) and (29). In these papers, the methodology presented includes both the scalar relativistic and SOC effects perturbatively, leading to many contributions. From the results, it is however immediately clear that SO coupling plays the essential role, and thus, for the purpose of analyzing the g-tensor contributions qualitatively, it is sufficient to include only the SOC dependent terms. In ref (15), we analyzed the contribution to the g-tensor, which includes the one-electron SO interaction in a linear fashion. We showed that for systems containing one heavy atom and for the purpose of qualitative analysis, it is sufficient to include only the SOC generated by a single heavy atom present in the system. In addition, if one integrates the spin degrees of freedom out of the expressions, the SOC is represented by three simple scalar operators . Here, *r*_*M*_ is the distance between the electron and nucleus *M*, *Z*^*M*^ is the nuclear charge of nucleus *M*, and  represents the *u*th component of the angular momentum operator centered on nucleus *M*. Then, the dominant contribution to the OZ term in eq 1 has the form
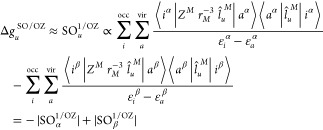
2and will from now on be referred to as SO/OZ. In eq 2, the indexes *i* and *a* denote occupied and vacant molecular orbitals, respectively. Here and in the following, we assume that the molecule is oriented so that the principal axis frame of the g-tensor coincides with the Cartesian coordinate system, and thus, Δ*g*_*u*_^*X*^ represents the contribution to the *u*th eigenvalue of the g-tensor. Because the numerators in eq 2 are positive—if one neglects interatomic contributions—and the denominators are always negative, the first (alpha) term on the right hand side (rhs) of the equation gives a negative contribution and the second (beta) term gives a positive contribution. For a more detailed analysis and discussion of the above expression, see ref (15). When neglecting spin polarization effects, *i.e.*, when utilizing the spin restricted (SR) methodology, eq 2 can be simplified to include only couplings involving either the singly occupied molecular orbital (SOMO) or the singly unoccupied molecular orbital (SUMO)
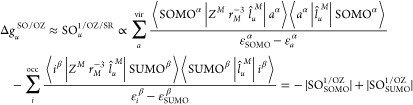
3

This expression is especially useful for the analysis of contributions to the g-shift induced by linear SO effects because, in contrast to eq 2, the number of contributions is greatly reduced, while the essence of the electronic interactions responsible for the g-shift is preserved. The equation is valid for a doublet system; however, the theory can easily be extended for higher spin states through summation over multiple singly occupied MOs.

The effect of second-order SO effects on the SZ contribution to the g-shift (denoted as SO^2^/SZ from now on) can be expressed using third-order PT, leading to the following equations^28,30^
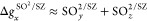
4
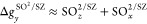
5
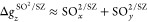
6
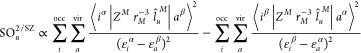
7

These expressions were presented in ref (28) for the first time in the framework of the spin-restricted methodology (see term *H*_SZ/SO_ in eq 19). We have implemented this contribution in the framework of spin-unrestricted DFT theory (see  in Section 6 of ref (30)) with the primary purpose of analyzing the dominant contributions from MSOs, and thus, we employed a few approximations^7^ to ease the chemical analysis (for a more detailed description, we refer the reader to the Methods section). In eq 7, the metal-centered one-electron operator  couples occupied (*i*) and vacant (*a*) one-component MSOs bearing opposite spins. The magnitude of the SO-induced coupling is divided by the square of the energy gap, focusing the interaction to energetically very close frontier MSOs. In eq 7, one may again neglect spin polarization effects to obtain expressions more suitable for the analysis of the contributions to the g-shift. The contributions arising from doubly occupied MSOs are cancelled out—*i.e.*, for each summand in the first term on the rhs of eq 7 (*i*^α^ ↔ *a*^β^), there exists one with the opposite sign in the second term (*i*^β^ ↔ *a*^α^)—and only couplings involving the SOMO and SUMO remain
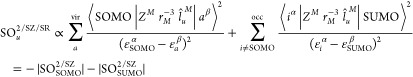
8

Both SOMO ↔ *a*^β^ and *i*^α^ ↔ SUMO couplings are of the type *i*^α^ ↔ *a*^β^, so all terms that do not subtract originate from the first sum in eq 7 and have a negative sign due to the squared  operator containing an imaginary unit. In contrast to the SO/OZ expression in eq 3, all contributions in the spin-restricted SO^2^/SZ expression of eq 8 are thus negative, and the contribution of quadratic SOC to the SZ g-shift is negative in general. This is the main theoretical result of this work. A positive SO^2^/SZ may occur only when spin polarization effects are bigger than the SOMO/SUMO contributions, which usually happens when the overall SO^2^/SZ contribution is small and thus not interesting. Closest to this result was the work of Bolvin,^22^ who applied a method based on the Gerloch and McMeeking formula^31^ and the ab initio two-step approach to a set of small symmetrical systems. To test the validity of the assumption of approximate cancelation in our spin-unrestricted scheme, we compared the g-shifts of selected compounds arising solely from couplings involving either the SOMO or SUMO with the full values and found them to differ by 3% at the maximum (see Table S1 in the Supporting Information). It is thus well justified to consider only couplings involving either the SOMO or SUMO in our case.

In our following analysis of the MSO contributions to the components of the g-shift, we refrain from analyzing SO^2^/OZ contributions, mainly because of their complexity (12 terms per each component of the g-shift)^30^ and also because their effect on the g-tensor is smaller than that of SO_*u*_^2/SZ^ (vide infra).

Note that in contrast to eq 3, where both Δ*g*_*u*_^OZ^ and SO_*u*_^1/OZ/SR^ depend on the same Cartesian component *u*, in eqs 4–6, the SO_*u*_^2/SZ^ term contributes to the orthogonal components of the g-shift. Therefore, the total contribution from the SO interaction to the g-shift analyzed in this work is determined by the sum of the various SO_*u*_^1/OZ^ and SO_*v*_^2/SZ^ contributions, where the final sign of the *u*th component of the g-shift results from the interplay of three contributions SO_*u*_^1/OZ^, SO_*v*_^2/SZ^, and SO_*w*_^2/SZ^, where *v* and *w* are both Cartesian components orthogonal to *u*.

In summary, the g-shift calculated with our PT-based tool is the sum of three terms
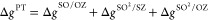
9

A brief comparison of contributions to the g-shift from the OZ and SZ operators obtained with the four-component method (eq 1) and PT (eq 9) is shown in Table S2.

## 3 Results and Discussion

The 4c approach used in this work currently represents the best available methodology for predicting the g-tensor of systems containing heavy elements within the DFT framework. It includes relativistic effects at a high level of precision using the Dirac–Coulomb Hamiltonian and utilizes a Kramers unrestricted Kohn–Sham determinant and noncollinear exchange–correlation functionals to account for spin polarization effects. However, 4c methods are not suitable for in-depth chemical analysis due to complicated expressions that go beyond the usual chemical intuition. In contrast, although PT expressions are more approximate than those of 4c, they are more suitable for analysis because all formulas are expressed using the familiar non-relativistic molecular orbitals. Therefore, in the following, we use the 4c methodology to obtain the correct quantitative description of the g-tensor and PT to supply a qualitative analysis of the underlying chemical concepts.

**Figure 1:**
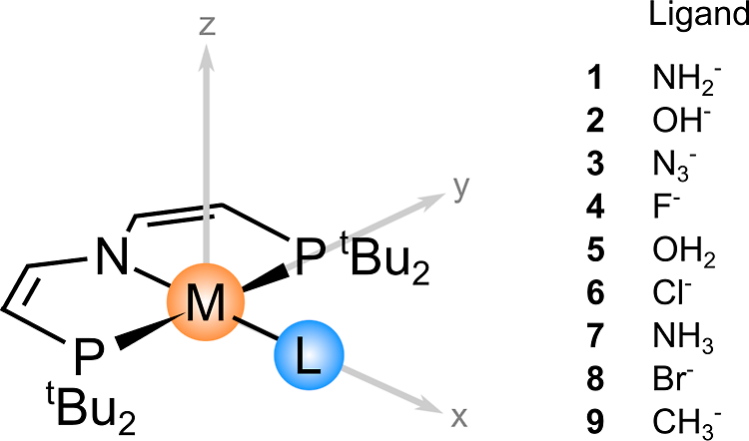
Structure of the M(II) d^7^ complexes (M = Ir and Rh) investigated and their orientation in the Cartesian coordinate system. The overall charge is zero except for complexes **5** and **7**, which have neutral ligands. Complexes **1**, **2**, **3**, **6**, and **7** as well as **Rh3** and **Rh6** have previously been synthesized and characterized.^32−36^

A summary of all of the complexes investigated in this work and their general structures is shown in Figure 1. Three coordination sites in these square-planar Ir complexes are occupied by a PNP ligand bearing protecting *tert*-butyl groups. The last site in the trans position relative to the N linking atom carries the variable ligand (L), which modulates the electronic structure of the paramagnetic center. In our previous work,^15^ we found higher-order contributions to be more pronounced in the Ir(II) system (complex **6** in the present work), so we now decided to focus on the Ir(II) oxidation state [see Table S3 in the Supporting Information for the magnitude of quadratic contributions in the Ir(IV) system]. This is also advantageous because multiple derivatives of the Ir(II) pincer complexes that differ only in the terminal ligand are known experimentally.^32−35^ We further extended the set of known complexes by modifying the terminal ligand in silico, which allowed us to investigate the mechanisms even more generally.

In the following sections, we first analyze the MSO contributions to the g-shift in complex **6** to establish a general mechanism. We then systematically explore the effect of different factors, which appear in eqs 3 and 8.

(i) The nuclear charge of the central metal atom.
(ii) The energy gaps between interacting orbitals.
(iii) The matrix elements, whose magnitude is related to the delocalization of the interacting orbitals.

The effect of nuclear charge is demonstrated by comparing the g-tensors of complexes **6** and **Rh6**. The influences of the energy gaps and matrix elements on the g-tensor are analyzed in the extended series of Ir complexes with a variable terminal ligand.

### 3.1 Molecular Orbital Analysis of SO Contributions to the g-Shift in Complex **6** (Ir–Cl)

**Figure 2:**
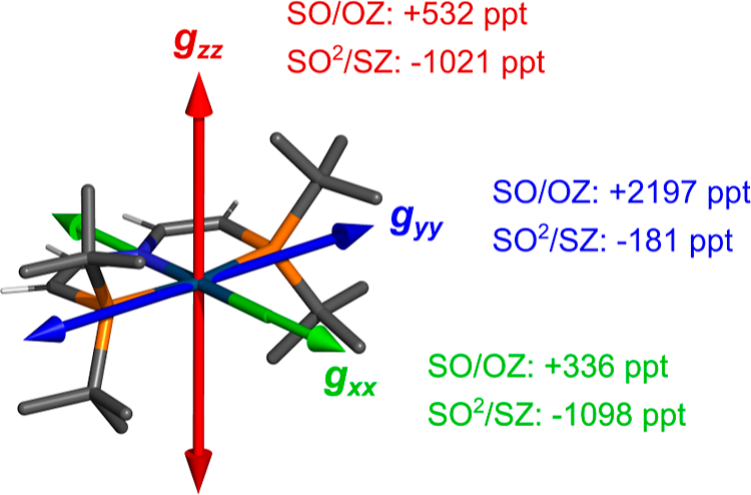
Orientation of the principal axes of the g-tensor in complex **6** from the 4c/PBE0/TZ/vac calculation and the contributions to the corresponding components of the g-shift from the SO/OZ and SO^2^/SZ terms calculated by using the PT/PBE/DZ/vac approach (see Section 5, Methods).

The orientation of the calculated principal axes of the g-tensor together with the contributions from the SO/OZ and SO^2^/SZ terms is shown in Figure 2. The analysis of the g-tensor of compound **6** in our previous work^15^ focused mainly on the largest g-shift component, Δ*g*_*y*_. While Δ*g*_*y*_ is indeed dominated by the SO/OZ contribution and inclusion of the higher-order term does not change it qualitatively, it is crucial for Δ*g*_*x*_ and Δ*g*_*z*_, where the SO^2^/SZ contribution is larger than that of SO/OZ.

**Figure 3:**
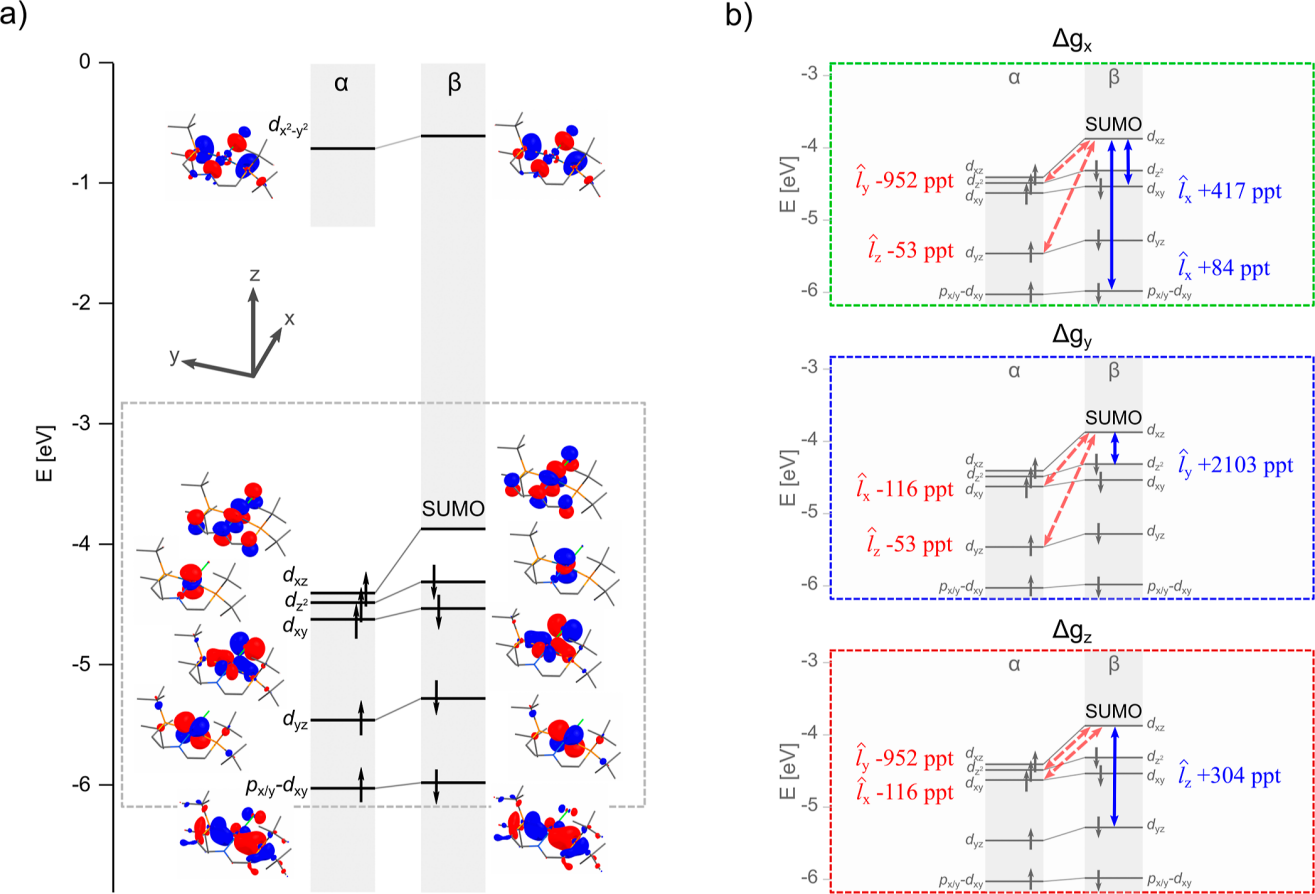
(a) Simplified MSO diagram calculated for compound **6** (Ir–Cl) with KS/PBE/DZ/vac and (b) portion of the diagram with highlighted occupied ↔ vacant couplings and their contributions to individual components of the g-shift. The component of the angular momentum operator involved in the integral (see Section 2, Theoretical Background) is given beside each contribution. All couplings that contribute at least 10% of Δ*g*_*u*_^term^ (where the term = SO/OZ or SO^2^/SZ) are included. Note that while the SO/OZ mechanism acts between MSOs with the same spin, the SO^2^/SZ mechanism facilitates couplings between α and β MSOs.

The MSO diagram of compound **6** is shown in Figure 3a. Its main features and the link to the SO/OZ contribution to the g-tensor were discussed quite thoroughly in our previous work;^15^ nevertheless, we will briefly repeat the main points. The SOMO/SUMO pair has the character of an Ir-based d_*xz*_ orbital with π-antibonding interactions to the p_*z*_-orbitals of the PNP and chloride ligands—π*(d_*xz*_–p_*z*_). As noted in the Theoretical Background section, to a very good approximation, the g-shift arises only from couplings that involve one of the SOMO/SUMO pair. A strong coupling via the SO/OZ mechanism between the vacant β-π*(d_*xz*_–p_*z*_) orbital, SUMO, and occupied nonbonding (NB) β-d_*z*^2^_ orbital, β-NB, dominates the Δ*g*_*y*_ component of the g-shift with a large positive contribution (+2103 ppt, cf. Figure 3b). Couplings of the SUMO with lower-lying β-d_*xy*_- and β-d_*yz*_-based MSOs also produce a positive g-shift in the cases of the remaining two components (+417 ppt for Δ*g*_*x*_ and +304 ppt for Δ*g*_*z*_, cf. Figure 3b). The SO/OZ mechanism is in fact incapable of producing a negative g-shift in complex **6** because it acts only through β ↔ β couplings; α ↔ α couplings are inefficient here as only one Ir-based d-orbital is present in the vacant space (), and it is far in energy from the SOMO. This is a typical situation for complexes late in the transition metal series.^1^

The SO^2^/SZ mechanism acts through couplings of α ↔ β MSOs. The most important SO^2^/SZ contribution (−952 ppt, cf. Figure 3b) arises from the coupling of the SUMO with the occupied NB orbital α-NB, *i.e.*, the opposite-spin partner of the MSO that generates the large SO/OZ contribution to Δ*g*_*y*_ (vide supra). This coupling is more efficient than the smaller SO/OZ contributions to Δ*g*_*x*_ and Δ*g*_*z*_ due to the smaller energy gap. The difference in the dominant mechanism for different components of the g-shift is linked directly to the switched directions in eqs 2 and 4–6. The large SO/OZ contribution to Δ*g*_*y*_ is transferred to the SO^2^/SZ contribution in the perpendicular directions *x* and *z*. It is interesting to note that due to the negative sign of the SO^2^/SZ contribution and the fact that it contributes primarily to different components of the g-shift than the positive SO/OZ term, its inclusion increases the anisotropy of the g-tensor.

If we were instead dealing with a complex with negative α ↔ α couplings dominating the linear SO/OZ term (*i.e.*, one that comes earlier in the transition series, with the electron configuration d^1^–d^5^), then the always negative SO^2^/SZ term would enhance rather than compete with the negative SO/OZ g-shift and would be expected to lower the anisotropy of the g-tensor instead of increasing it. In the Supporting Information, we briefly show two examples (d^1^ complex OsOF_5_, Figure S1, and d^5^ Ir(IV) pincer complex, Table S3), where the SO/OZ mechanism acts primarily through negative α ↔ α couplings.

### 3.2 Role of the Heavy Atom

To explore the effect of the central metal atom (d^7^) on the g-tensor, we selected the Rh(II) analogue of **6**. Linear and quadratic SO contributions to the individual components of the g-shift calculated for **Rh6** and **6** with PT are summarized in Table 1.

**Table 1:** Linear and Quadratic SO Contributions to the Individual Components of the g-Shift (in ppt) Obtained from PT for the **Rh6** Analogue^36^ and the Ir Compound **6** with PT/PBE/DZ/vac^a^

compound **6**	**Rh**	Δ*g*_*x*_	Δ*g*_*y*_	Δ*g*_*z*_	**Ir**	Δ*g*_*x*_	Δ*g*_*y*_	Δ*g*_*z*_
	SO/OZ	268	**1134**	175	SO/OZ	532	**2197**	336
	SO^2^/OZ	80	–74	–60	SO^2^/OZ	410	–321	–292
	SO^2^/SZ	–**177**	–36	–**193**	SO^2^/SZ	–**1021**	–181	–**1098**
	full PT	171	1024	–78	Full PT	–79	1695	–1053
	4c	–391	1067	–602	4c	–134	1299	–641
	EXP	–179	1243	–318	EXP	–42	1348	–482

a Relativistic four-component values (4c/PBE0/TZ/vac) and experimental data are listed in the last two lines.

The most significant contributions arise from the orbital couplings that have been described in Section 3.1. Apart from the different nuclear charge *Z* (and thus the different strength of the SO interaction), the g-shift might differ between the two complexes due to different energy gaps between the relevant orbitals or different matrix elements in the numerator, see eqs 2 and 7. However, the analysis of these two characteristics summarized in Table 2 indicates that they are quite similar in **Rh6** and **6** and the observed difference in the various contributions to the g-tensor components is thus mostly governed by *Z*.

**Table 2:** Characteristics of the MSOs That Produce the Dominant Coupling in Compounds **Rh6** and **6**^a^

compound **6**	Δ*E*	Ir d_*z*^2^_ β-NB	Ir d_*xz*_ SUMO	Cl p_*z*_ SUMO	*r*_M–L_	Δ*g*_*y*_^SO/OZ^ β-NB ↔ SUMO
Rh	0.39	77	54	10	231	**1089**
Ir	0.44	72	48	11	232	**2103**

a The energy gap (Δ*E* in eV), contributions (in %) of iridium d-AOs and chlorine p-AOs in the SUMO, β-π*(d_*xz*_–p_*z*_), and its coupling partner β-NB, β-, M–Cl bond length (*r*_M–L_ in pm, from structures optimized in vacuum), and the resulting Δ*g*^SO/OZ^ (in ppt) are shown. Mulliken population analysis was carried out on the KS/PBE/DZ/vac wavefunction.

As shown in Table 1, the Δ*g*_*y*_ component is governed by the linear SO/OZ contribution and amounts to roughly 1100 ppt in compound **Rh6**, approximately half of that of the Ir compound **6** (∼2200 ppt). In contrast, the SO^2^/SZ contribution to Δ*g*_*x*_ and Δ*g*_*z*_ is more than four-fold larger in the Ir(II) compound **6** than in **Rh6**. This trend can be easily understood when one realizes that the SO/OZ contribution depends on the nuclear charge linearly, while SO^2^/SZ contributes quadratically, see eqs 2 and 7. The effect of nuclear charge is thus more important for the quadratic SZ mechanism than for the linear OZ mechanism. Therefore, generally speaking, the relative role of the SO^2^/SZ contribution increases with increasing nuclear charge.

### 3.3 Role of the Terminal Ligand in a Series of d^7^ Ir(II) Complexes

**Figure 4:**
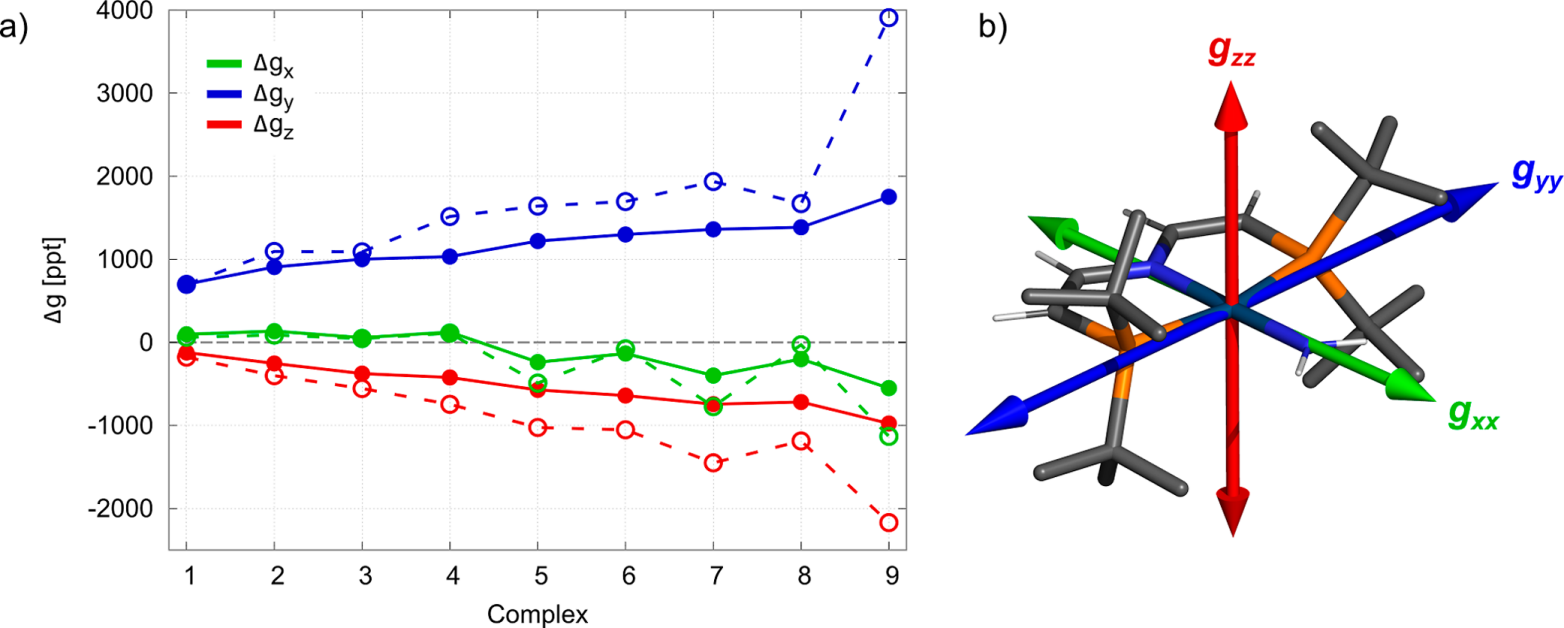
(a) Trend of the g-shift components along a series of Ir(II) compounds calculated with 4c/PBE0/TZ/vac (full line) or PT/PBE/DZ/vac (dashed line, eq 9). The ligand series is ordered according to the largest component Δ*g*_*y*_ in the 4c calculation. For comparison of calculations with available experimental values and the effect of the solvent, see Tables S4 and S5, respectively. (b) Orientation of the principal axes of the g-tensor with respect to the molecular geometry of complex **1** (Ir–NH_2_) from 4c/PBE0/TZ/vac. The orientation does not change qualitatively throughout the series.

When investigating the g-tensor of compound **6**, we also gathered the available experimental data on related compounds and noticed an interesting trend of the g-tensor anisotropy varying with the terminal ligand. This experimental trend was reproduced by four-component calculations (see Table S4 in the Supporting Information), which encouraged us to investigate its origins more deeply. Because we are interested in analyzing the trend using PT, it is important to establish whether PT qualitatively reproduces the trend given by four-component calculations. Figure 4a shows that this is the case for the extended series of compounds **1–9**. The anisotropy varies along the series of complexes due to a simultaneous increase in Δ*g*_*y*_ (blue, from +701 ppt in **1** to +1753 ppt in **9**) and decrease in Δ*g*_*z*_ (red, from −122 ppt in **1** to −977 ppt in **9**). We will now investigate this trend by decomposing the g-shift from PT to see how each of the contributions varies in the series of complexes.

**Figure 5:**
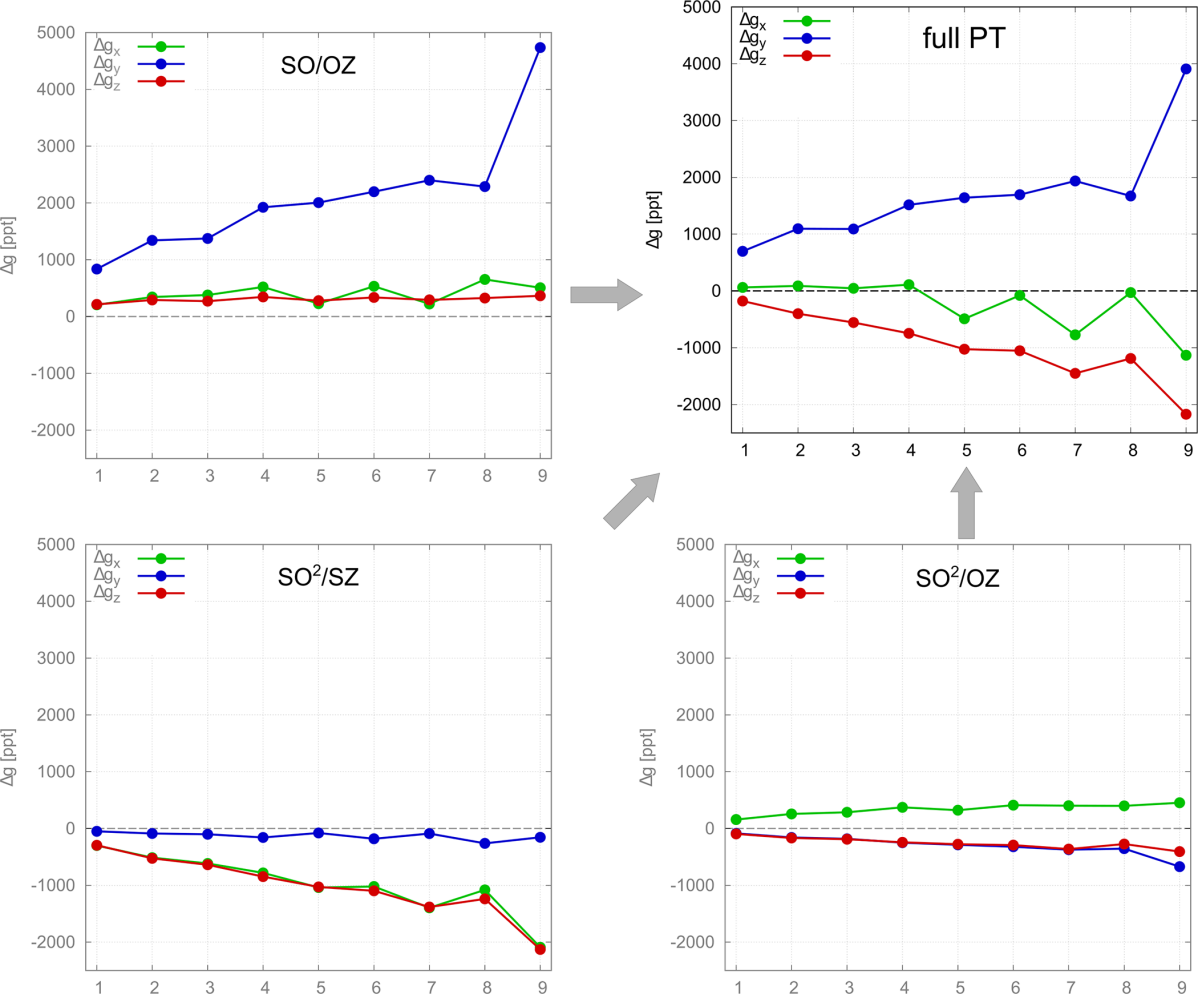
Decomposition of the g-shift components obtained with PT (PT/PBE/DZ/vac) into contributions from the SO/OZ, SO^2^/SZ, and SO^2^/OZ mechanisms along a series of Ir(II) compounds. The full value (PT) is equal to the sum of the individual contributions as defined in eq 9. For numerical data, see Table S6.

The decomposition of the g-shift in the series of compounds **1–9** is shown in Figure 5. The SO/OZ contribution to Δ*g*_*y*_ varies substantially in this series of compounds and is sufficient to describe the observed trend for this component. In contrast, the SO/OZ contribution remains relatively constant for Δ*g*_*x*_ and Δ*g*_*z*_ and yields the wrong sign (positive). The variation of the SO^2^/SZ contribution to Δ*g*_*x*_ and Δ*g*_*z*_ closely follows the variation of Δ*g*_*y*_^SO/OZ^—a larger (more positive) SO/OZ contribution to Δ*g*_*y*_ implies larger (more negative) SO^2^/SZ contributions to Δ*g*_*x*_ and Δ*g*_*z*_. This is again related to the switched directions in eq 3 for the SO/OZ mechanism and eqs 4 and 5 for the SO^2^/SZ mechanism. Both the SO/OZ and SO^2^/SZ mechanisms are thus responsible for the variation of anisotropy in the series of compounds **1–9**.

Figure 5 also includes the SO^2^/OZ term and allows us to describe its overall effect. The SO^2^/OZ term splits apart Δ*g*_*x*_ and Δ*g*_*z*_, which the two larger contributions do not distinguish, by slightly increasing the former and slightly lowering the latter. It also slightly lowers Δ*g*_*y*_. We believe that the relationship between the SO^2^/OZ term and the symmetry and electronic structure of the complexes, which underlies the clear trend seen in Figure 5, could be found by approximations similar to those used to understand the two larger contributions. Nevertheless, we will not analyze the SO^2^/OZ term any further as its complexity hinders a meaningful interpretation of the MSO contributions (see the Theoretical Background section and ref (30)). Luckily, it can be seen in Figure 5 that it is the smallest of the three contributions (for any given component of the g-shift, one of the other two terms is always more important) and varies the least throughout the series.

### 3.4 Effect of the Energy Denominator

It is worthwhile to focus first on the difference between complexes **1** (NH_2_^–^) and **7** (NH_3_), whose structures differ only in the presence or absence of one proton but which nevertheless differ significantly in anisotropy. The decomposition of the SO contributions to the g-shift up to the second order for the two systems is shown in Table 3.

**Table 3:** Linear and Quadratic SO Contributions to the Individual Components of the g-Shift (in ppt) Obtained from PT/PBE/DZ/vac for Compounds **1** (NH_2_^–^) and **7** (NH_3_)

**1** (NH_2_^–^)	Δ*g*_*x*_	Δ*g*_*y*_	Δ*g*_*z*_	**7** (NH_3_)	Δ*g*_*x*_	Δ*g*_*y*_	Δ*g*_*z*_
SO/OZ	206	**836**	214	SO/OZ	222	**2399**	294
SO^2^/OZ	157	–87	–96	SO^2^/OZ	402	–373	–362
SO^2^/SZ	**–300**	–50	**–296**	SO^2^/SZ	**–1396**	–90	**–1383**
full PT	62	698	–178	full PT	–772	1936	–1451

A striking difference in the magnitude of the SO/OZ contribution is found for the Δ*g*_*y*_ component, which is larger by 1563 ppt in **7** (2399 ppt) than in **1** (836 ppt). This is due to the different strength of the β-NB ↔ SUMO coupling (1560 ppt of the difference arises exclusively from this main coupling). The effect propagates to the perpendicular g-shift components through the SO^2^/SZ mechanism, whose contribution to Δ*g*_*x*_ and Δ*g*_*z*_ is approximately 1100 ppt more negative in **7** than in **1** (1077 ppt of the difference arises from the main α-NB ↔ SUMO coupling). From now on, we will focus only on these two most important couplings.

**Figure 6:**
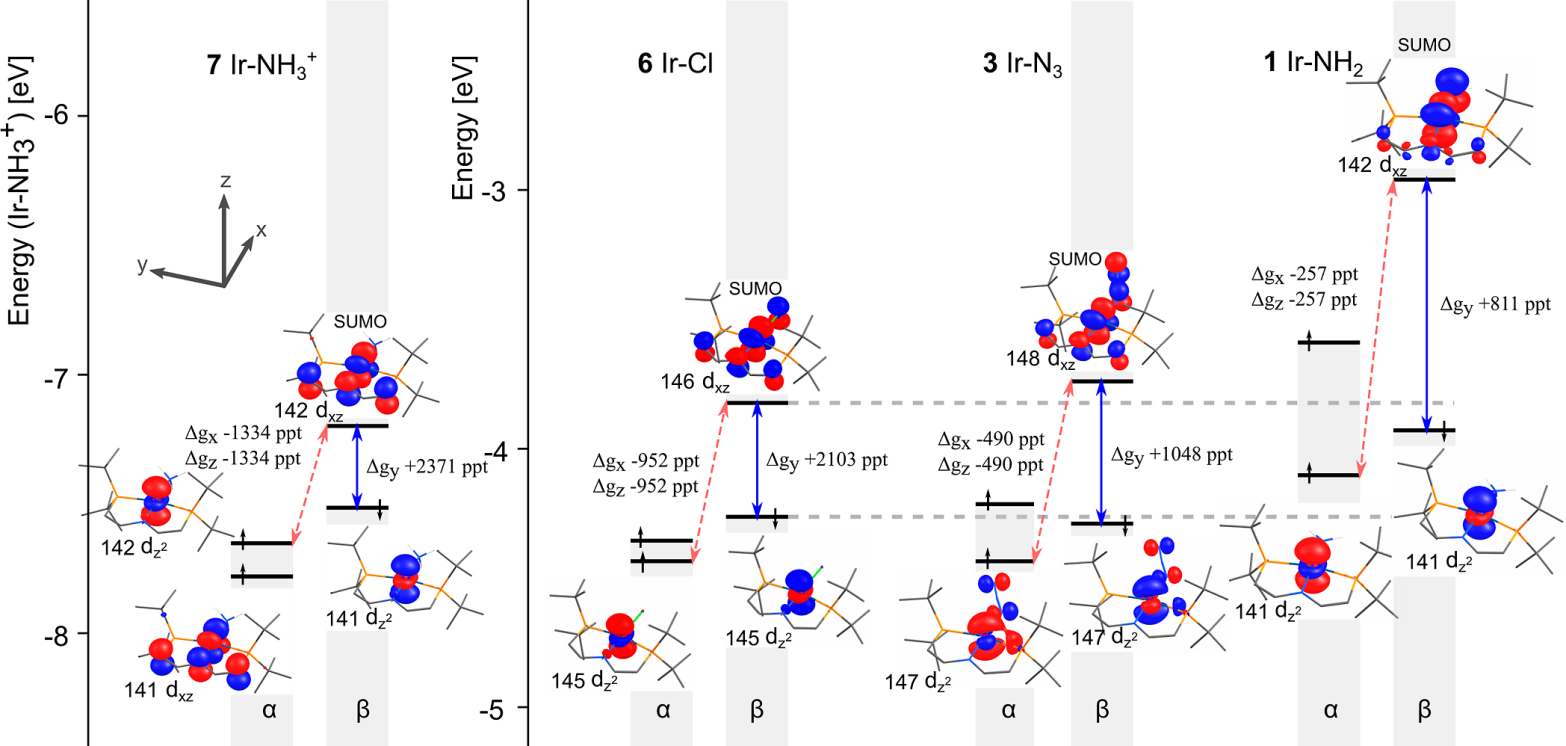
Energy diagram showing selected frontier MSOs calculated for complexes **7**, **6**, **3**, and **1** at the nonrelativistic (KS/PBE/DZ/vac) level. Note the energy offset due to the neutral character of the NH_3_ ligand and the different overall charge of the complex. The values of the g-shift components indicate the contributions of the labeled MSO couplings arising from the SO/OZ term (β ↔ β couplings) or the SO^2^/SZ term (α ↔ β couplings). All indicated couplings arise from the *y* component of the operators.

The abovementioned differences in the magnitudes of the g-shifts are principally caused by variations in the energy gaps between the interacting α/β-NB orbital and SUMO due to different terminal ligands. A simplified energy level diagram highlighting only these key MSOs for compounds **1**, **3**, **6**, and **7** is shown in Figure 6. As the SUMO is antibonding with respect to the π-type interaction with ligand p_*z*_-orbitals, stronger metal–ligand (M–L) π interaction destabilizes it. The d_*z*^2^_-based MSO is less affected because of its mostly NB character (the slight differences arise from σ-type interactions).

The energy gap between the β-NB orbital and SUMO in **7**, where the p-orbitals of L are not involved in the M–L bonding, is only 0.32 eV (KS/PBE/DZ). On the contrary, in the closely related complex **1**, a p “lone pair” on N remains available for interaction with the d_*xz*_ of Ir. As a result, the SUMO is more destabilized in **1**, the energy gap is larger (0.97 eV, KS/PBE/DZ), and both Δ*g*_*y*_ and Δ*g*_*z*_ are smaller in magnitude. Also note that the changes in the energy gap have a larger effect on the SO^2^/SZ mechanism than on the SO/OZ mechanism as the energy difference in the denominator in eq 8 is squared, while in eq 3, the dependence is only linear.

### 3.5 Effect of the Matrix Elements: Delocalization of the Interacting Orbitals

In addition to the nuclear charge and energy gaps discussed in the previous sections, the magnitudes of g-shifts are influenced by the values of the matrix elements in the numerator (see eqs 2 and 7). Because the SOC is largely generated by the central heavy atom in our systems, the size of the numerators is given to a large extent by localization of the interacting molecular orbitals at the central Ir.

**Figure 7:**
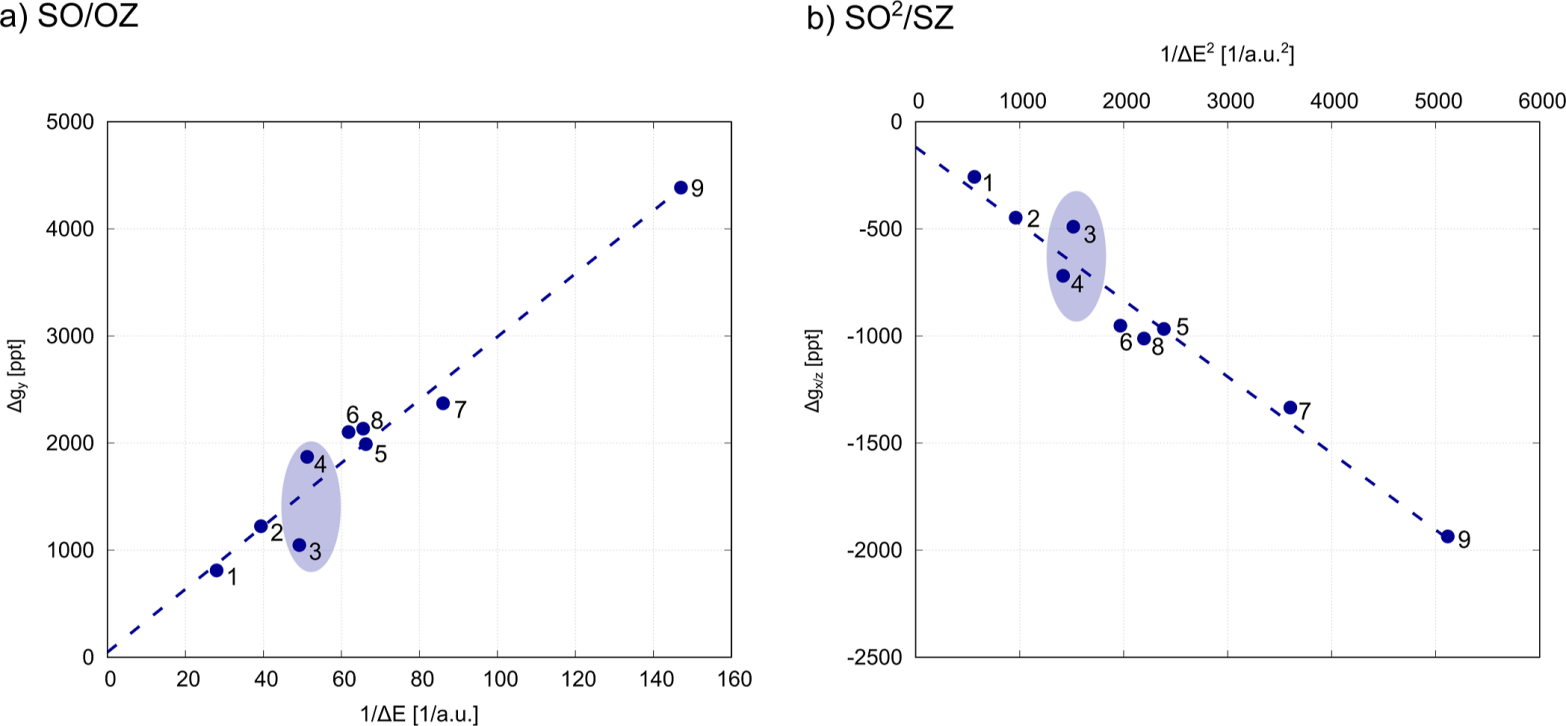
Dependence of the Δ*g* arising from the largest coupling between a pair of MSOs (PT/PBE/DZ/vac) through the (a) SO/OZ term (β-NB ↔ SUMO) or (b) SO^2^/SZ term (α-NB ↔ SUMO) on the energy separation of the two MSOs, 1/Δ*E* or 1/Δ*E*^2^ (see the Theoretical Background section for the relevant equations).

Figure 7 shows Δ*g*_*y*_ and Δ*g*_*x*/*z*_ arising from the dominant NB ↔ SUMO coupling through either the SO/OZ or SO^2^/SZ mechanism plotted against 1/Δ*E* and 1/Δ*E*^2^, respectively. These dependences are expected to be linear in the case of a similar magnitude of the matrix elements in the numerator as the reciprocal energy gaps directly enter the calculation of Δ*g*_*u*_. This turns out to be the case, pointing to the decisive role of varying energy gaps in explaining the trends in the series of complexes investigated. Nevertheless, irregularities do appear in the dependence. Most notably, the values of the g-shift for compounds **3** (N_3_^–^) and **4** (F^–^) differ much more than the similar magnitudes of the energy gaps would suggest. We are thus motivated to analyze the interacting orbitals in order to understand the origin of this discrepancy.

We collected the contributions of the iridium d-AOs to the NB orbital and SUMO in complexes **3** and **4** from the Mulliken population analysis seen in Table 4. The percentage of the Ir d_*xz*_-AO in the SUMO is affected by delocalization to the ligand. From Table 4, it is clear that the SUMO of **3** is less localized on Ir (42%) than the SUMO of **4** (52%). This difference would, by itself, result in lower magnitudes of the matrix elements and thus smaller SO/OZ and SO^2^/SZ contributions to the g-shift in **3**.

**Table 4:** Energy (in eV) and Contributions (in %) of Ir d–AOs to MSOs (NB and SUMO) Whose Coupling Produces the Largest Δ*g*^SO/OZ^ and  (in ppt)^a^

	MSO ↔ MSO*	Δ*E*	β-NB	SUMO	^Δ*g*^SO/OZ^^
**3 (N**_**3**_**)**	β-NB ↔ SUMO	**0.55**	**60**	**42**	**1048**
	β-(NB-1) ↔ SUMO	0.93	11	42	277
**4 (F)**	β-NB ↔ SUMO	**0.53**	**70**	**52**	**1871**

		Δ*E*	α-NB	SUMO	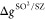
**3 (N**_**3**_**)**	α-NB ↔ SUMO	**0.70**	**56**	**42**	**–490**
	α-(NB-1) ↔ SUMO	1.04	14	42	–72
**4 (F)**	α-NB ↔ SUMO	**0.72**	**69**	**52**	**–719**

a Mulliken population analysis was carried out at the KS/PBE/DZ level.

It is, however, worth noting that also the amount of d_*z*^2^_-AO in the NB orbital, which contributes the largest coupling, is smaller in **3** (60% in the β space and 56% in the α space) than in **4** (70% in the β space and 69% in the α space). This might seem strange as the  is considered to be nonbonding and it is hard to imagine that it could be delocalized. The difference arises from symmetry. While the symmetry of complex **4** is approximately *C*_2*v*_, the bent N_3_^–^ ligand in **3** (in the plane of the Ir–PNP) causes lowering of the symmetry to *C*_*s*_. In *C*_*s*_, d_*z*^2^_ can interact with a lower-lying orbital of d_*xy*_ symmetry. This mixing produces an additional occupied MSO with a non-negligible metal d_*z*^2^_–AO admixture; in other words, part of the d_*z*^2^_–AO escapes into a lower-lying orbital. Coupling of this lower-lying MSO with the SUMO also contributes to Δ*g*_*y*_^SO/OZ^ and  but less effectively due to the larger energy gap (see Table 4).

## 4 Conclusions

In this work, we have performed a systematic study of the electronic g-tensor of square-planar Ir and Rh complexes employing the four-component DFT methodology to obtain the best theoretical results and the more approximate third-order PT for performing detailed molecular orbital analysis. In particular, we have implemented the third-order PT to analyze the effect of quadratic SO interaction on the g-tensor. Some of our most general observations are worth summarizing.

(i) The SO^2^/SZ contribution, which was the focus of this work, is generally negative. This was shown by introducing a “restricted” approximation to our spin-unrestricted equations.
(ii) A large SO/OZ contribution to one principal component is transferred through the SO^2^/SZ mechanism to a large negative contribution for perpendicular components.
(iii) If the SO/OZ contribution is positive (*i.e.*, it yields a large *g*_33_ via β ↔ β couplings in late transition metal compounds), SO^2^/SZ increases the anisotropy of the g-tensor by contributing a large negative g-shift to *g*_11_ and *g*_22_ (using the convention *g*_11_ < *g*_22_ < *g*_33_).
(iv) If the SO/OZ contribution is negative (*i.e.*, it yields a small *g*_11_ via α ↔ α couplings in early transition metal compounds), SO^2^/SZ decreases the anisotropy of the g-tensor by contributing a large negative g-shift to *g*_22_ and *g*_33_.

Apart from these general rules, we analyzed the effects of different factors that influence the magnitude of the resulting g-shift, namely, the nuclear charge *Z* of the metal, the localization of the orbitals on the central atom, and the energy gaps between these orbitals. In the investigated series of Ir d^7^ compounds, the most decisive factor for the anisotropy of the g-tensor turned out to be the stabilization/destabilization of the π antibonding SUMO influenced by the chemical character of the ligand, which determines its energetic separation from the NB d_*z*^2^_ orbital, whose coupling with the SUMO produces large SO/OZ and SO^2^/SZ contributions to the g-shift.

We believe that our study can provide a better qualitative understanding of EPR spectra of heavy transition metal systems in the language of MSOs, which is intuitive for chemists. Our results could potentially contribute to a better understanding of the paramagnetic NMR spectra of such compounds as the g-tensor is directly linked to hyperfine NMR shift.

## 5 Methods

### 5.1 Preparation of the Molecular System

The geometries of the transition metal complexes studied were obtained either from a crystallographic database or created by in silico modification of the existing structures in the cases of derivatives that are not experimentally known and subsequently fully optimized at the unrestricted PBE0^37^/def2TZVPP + ECP level in vacuum or using the IEFPCM model of solvent in the Gaussian16 program.^38^ The character of the local minimum of all obtained structures was verified using vibrational analysis. The degree of spin contamination was negligible (maximum value of  for compound **7**). The default convergence criteria (maximum force 0.00045 a.u. and maximum displacement 0.0018 Å) were applied.

### 5.2 Calculation of the g-Tensor

All g-tensors were calculated first at the four-component (4c) level in the program ReSpect (version 5.2.0, November 2020)^39,40^ by employing the Dirac–Kohn–Sham Hamiltonian with the PBE0 functional and Dyall-VDZ/VTZ^41^ and iglo-II/III basis sets^42,43^ for the central metal and ligand atoms, respectively (method labeled 4c/PBE0/DZ and 4c/PBE0/TZ). Here, labels DZ and TZ stand for the double- and triple-zeta basis set quality, respectively. As the iglo-III basis set is not defined for Br, we used Dyall-VTZ for this atom in the TZ calculation. All basis sets in the 4c calculations have been utilized in the uncontracted form. The noncollinear form of the exchange–correlation potential used in this work is specified in Table 1 in ref (44). The g-values obtained were compared with experimental values (if available).^32−34^ The effect of the CPCM model in ReSpect^45^ was tested as a way to account for the frozen solution environment, but solvent effects were modest and not crucial to reproduce the trend of varying anisotropy (see Table S5). Because the extended set includes compounds that have not yet been synthesized or characterized by EPR, we performed all subsequent calculations in vacuum (vac). As the PT analysis imposes limitations on the functional (only GGA) and basis set (only DZ), we checked how these aspects influence the calculated g-tensors at the 4c level. Whereas an increase in basis set quality (from DZ to TZ, Table S7) reduces the anisotropy (the average decrease in g-tensor span is ∼10%), inclusion of the exact exchange (PBE vs PBE0, Table S8) enlarges the anisotropy substantially (the average increase in the span is ∼30%). The relative trend of the g-tensor components along the series of complexes is preserved by all methods.

The analysis of the PT expressions for the diagonal components of the g-tensor was based on a non-relativistic ansatz and calculated at the KS/PBE/DZ level of theory in vacuum. The studied molecules were reoriented into the principal axis system of the g-tensor calculated at the 4c level prior to the PT analysis. Molecular orbitals were rendered in the program Chemcraft.^46^

### 5.3 Analysis of the Linear and Quadratic SO Contributions to the g-Tensor

To analyze SO effects in the g-tensor calculations, we have implemented the perturbation expressions for the first- and second-order SO contributions to the g-tensor (denoted SO_*u*_^1/OZ^ and SO_*u*_^2/OZ^ + SO_*u*_^2/SZ^, respectively) in the program ReSpect.^40^ For this purpose, we utilized second- and third-order PT; see, for example, refs (10)(47), and (48). A detailed description of the theory and implementation of the SO_*u*_^1/OZ^ and SO_*u*_^2/OZ^ + SO_*u*_^2/SZ^ contributions is presented in ref (30). To make the chemical analysis of  feasible, we have made approximations that result in eqs 4–7. We consider only one-electron SO interaction, *i.e.*, we neglect all two-electron (2e) SO effects. Because these effects are relatively more important for the g-tensors of compounds containing light elements,^12^ the analysis presented in this work is best applied to heavy-element compounds. Because the systems studied in this work contain a single heavy element, we approximate the operators that represent the one-electron SO operator as  with *Z*^*N*^ being the nuclear charge of the *N*th nucleus. We use operators that do not contain the Pauli matrices because we have reduced the working expressions eqs 2–8 to the one-component form by integrating out the spin degrees of freedom. As a result, the SO operator is represented by three operators. We choose the position of the heavy atom *M* as the gauge origin for the angular momentum operator to reduce the overall basis set requirements of the g-tensor calculations. This allows us to employ a relatively small DZ basis and still preserve trends predicted by the 4c calculations, while, at the same time, the number of significant MO couplings is considerably reduced compared to calculations obtained with a TZ basis. Finally, the MSO analysis is greatly simplified if one neglects contributions from the Hartree–Fock (hf) and exchange–correlation (xc) kernels (both first- and second-order). Especially in the case of the hf kernel, these effects might not be negligible. To mitigate, we perform the analysis using pure DFT functionals (*i.e.*, functionals without exact exchange) because xc second-order kernels are usually smaller than hf. First, this allows us to avoid considering the first-order kernel contributions that vanish because first-order SO-induced changes in charge and spin densities are zero. Second, the importance of second-order kernels is decreased and even if they are neglected, the analysis of chemical concepts is reasonable because the remaining one-electron contributions (eqs 4–7) still determine the chemical trends in the calculated results. For a further discussion of the MSO analysis, see the Methods sections in refs (8) and (15).

## Supporting Information Available

Additional theoretical details, calculated g-tensors, simplified molecular orbital diagram for the analysis of the g-tensor of OsOF_5_, g-tensor in the d^5^Ir(IV) complex, and analyses (PDF)
Examples of input/output files for the 4c–ReSpect and PT calculations (ZIP)

## Notes

Geometry optimization input files and Cartesian coordinates of optimized geometries are available in the ioChem-BD repository^49^ and can be accessed via https://iochem-bd.bsc.es/browse/handle/100/251529.
