# Supplementary material for: Quadratic Spin–Orbit Mechanism of the Electronic g-Tensor
Source: J Chem Theory Comput. 2023 Mar 10;19(6):1765–76. doi: 10.1021/acs.jctc.2c01213 (PMC10061661; doi:10.1021/acs.jctc.2c01213)
Supplement: Supplementary file 1 — ct2c01213_si_001.pdf [file ct2c01213_si_001.pdf]

SUPPORTING INFORMATION FOR

Quadratic Spin-Orbit Mechanism of the Electronic g-Tensor

Petra Pikulová,[†,‡] Debora Misenkova,[∆] Radek Marek,[†,‡] Stanislav Komorovsky,*[∆] Jan Novotný*[†,‡,∆]

[†] *CEITEC – Central European Institute of Technology, Masaryk University, Kamenice 5, CZ-62500 Brno, Czechia*

[‡] *Department of Chemistry, Faculty of Science, Masaryk University, Kamenice 5, CZ-62500 Brno, Czechia*

[∆] *Institute of Inorganic Chemistry, Slovak Academy of Science, Dúbravská cesta 9, SK-84536 Bratislava, Slovakia*

*Emails: S.K. stanislav.komorovsky@savba.sk, J.N. jan.novotny@ceitec.muni.cz

Decomposition of the g-tensor from four-component theory

In this work we decompose the four-component expressions for the calculation of the g-tensor (presented in Refs. 1,2) as follows

$$\boldsymbol{g} = g_e\mathbf{1} + \Delta\boldsymbol{g}^{\mathrm{SZ}} + \Delta\boldsymbol{g}^{\mathrm{OZ}} + \Delta\boldsymbol{g}^{\mathrm{REL}},$$

$$\Delta g_{uv}^{\mathrm{SZ}} = \frac{g_e}{2}\frac{1}{S}\,\mathrm{Re}\left\{\mathrm{Tr}\left[\boldsymbol{\Lambda}_u^{\mathrm{SZ}}\boldsymbol{P}^{\mathrm{LS}}(\vec{J}_v)\right]\right\} - g_e\delta_{uv},$$

$$\Delta g_{uv}^{\mathrm{OZ}} = \frac{g_e}{2}\frac{1}{S}\,\mathrm{Tr}\left[\boldsymbol{\Lambda}_u^{\mathrm{OZ}}\boldsymbol{P}^{\mathrm{LS}}(\vec{J}_v) + \boldsymbol{\Lambda}_u^{\mathrm{OZ}}\boldsymbol{P}^{\mathrm{SL}}(\vec{J}_v)\right],$$

$$\Delta g_{uv}^{\mathrm{REL}} = \frac{g_e}{2}\frac{1}{S}\,\mathrm{Re}\left\{\mathrm{Tr}\left[\boldsymbol{\Lambda}_u^{\mathrm{REL}}\boldsymbol{P}^{\mathrm{SL}}(\vec{J}_v) - \boldsymbol{\Lambda}_u^{\mathrm{REL}}\boldsymbol{P}^{\mathrm{LS}}(\vec{J}_v)\right]\right\},$$

$$(\boldsymbol{\Lambda}_u^{\mathrm{SZ}})_{\lambda\tau} = \langle\boldsymbol{X}_\lambda|\boldsymbol{\sigma}_u|\boldsymbol{X}_\tau\rangle, \qquad \mathbf{X}_\lambda = \mathbf{1}_{2\times2}\chi_\lambda,$$

$$(\boldsymbol{\Lambda}_u^{\mathrm{OZ}})_{\lambda\tau} = \left\langle\boldsymbol{X}_\lambda\middle|\frac{1}{2}(\vec{r}_G\times\vec{p})_u\mathbf{1}_{2\times2}\middle|\boldsymbol{X}_\tau\right\rangle,$$

$$(\boldsymbol{\Lambda}_u^{\mathrm{SZ}})_{\lambda\tau} = \left\langle\boldsymbol{X}_\lambda\middle|\frac{i}{2}\,[\vec{r}_G\times(\vec{p}\times\vec{\boldsymbol{\sigma}})]_u\middle|\boldsymbol{X}_\tau\right\rangle.$$

Here Re{c} denotes the real part of the complex number c, Tr[$\boldsymbol{A}$] represents the trace of matrix $\boldsymbol{A}$, g_e is the absolute value of the electron spin g-factor ($g_e \approx 2.002319$), χ_λ is a scalar basis function, $\vec{\boldsymbol{\sigma}}$ is a vector composed of Pauli matrices, $\vec{p}$ represents the momentum operator, and $\vec{r}_G = \vec{r} - \vec{r}_0$ with $\vec{r}$ and $\vec{r}_0$ being the position of the electron and gauge origin vector, respectively. The density matrices $\boldsymbol{P}^{\mathrm{LS}}$ and $\boldsymbol{P}^{\mathrm{SL}}$ are defined, for example, in Ref. 2 The theory for relativistic calculations of the g-tensor that includes spin-orbit interaction variationally (self-consistently)[3] requires the calculation of three Kohn-Sham determinants, each with a different orientation of the magnetization vector $\vec{J}_v$. Finally, S denotes the fictitious (effective) spin of the system, where the formal multiplicity of the system, $2S+1$, represents the number of populated states under the experimental conditions.

Table S1. Validity of the SOMO-SUMO approximation for the investigated SO/OZ and SO^2/SZ contributions to the components of the g-shift (Δg in ppt) calculated with PT analysis.

				Δg_x	Δg_y	Δg_z
**1**	Ir-NH_2	SO/OZ	SOMO+SUMO	200	838	213
			total	206	836	214
		SO^2/SZ	SOMO+SUMO	-300	-51	-295
			total	-300	-50	-296
**3**	Ir-N_3	SO/OZ	SOMO+SUMO	371	1375	265
			total	378	1374	270
		SO^2/SZ	SOMO+SUMO	-617	-104	-639
			total	-615	-102	-639
**6**	Ir-Cl	SO/OZ	SOMO+SUMO	522	2197	327
			total	532	2197	336
		SO^2/SZ	SOMO+SUMO	-1024	-183	-1098
			total	-1021	-181	-1098
**7**	Ir-$NH_3^{(+)}$	SO/OZ	SOMO+SUMO	216	2390	286
			total	222	2399	294
		SO^2/SZ	SOMO+SUMO	-1399	-92	-1384
			total	-1396	-90	-1383

Table S2. Individual components of the Δg (in ppt) of compound **6** separated into contributions from the orbital-Zeeman and spin-Zeeman mechanisms from perturbation theory (OZ is the sum of SO/OZ and SO^2/OZ terms, SZ corresponds to SO^2/SZ) and the four-component calculation (PBE/DZ/vac).

	Δg_x		Δg_y		Δg_z	
	OZ	SZ	OZ	SZ	OZ	SZ
**PT**	942	-1021	1876	-181	44	-1098
**4c**	488	-476	1087	-126	119	-568

Analysis of the g-tensor in the d^1 compound $OsOF_5$

An analysis of the g-tensor of $OsOF_5$ with a simplified molecular orbital diagram is shown in Figure S1. This compound features osmium in the high oxidation state of VII, it is thus a d^1 complex. The unpaired electron occupies a d_{xy}-based SOMO; the remaining *d* orbitals of the metal are vacant. In such an electronic situation, the SO/OZ mechanism acts *via* α↔α couplings and produces a negative g-shift.[4] The linear SO/OZ term dominates components $\Delta g_\perp$ (Δg_x and Δg_y) with a large negative contribution of -417 ppt (-450 ppt originates from couplings of the SOMO with the degenerate vacant α-d_{xz} or α-d_{yz} for Δg_x and Δg_y, respectively). The overall SO/OZ contribution to $\Delta g_\parallel$ (Δg_z) is a balance between a negative SOMO ↔ α-$d_{x^2-y^2}$ coupling (involving the unoccupied σ-antibonding orbital derived from $d_{x^2-y^2}$) and the positive β-$d_{x^2-y^2}$ ↔ SUMO coupling (involving the occupied σ-bonding orbital derived from $d_{x^2-y^2}$) and turns out positive (this sign is inconsistent with the experimental data and 4c calculations[2,5]). The SO^2/SZ term transfers both of the large couplings facilitated by operators of $\hat{l}_x$ and $\hat{l}_y$ symmetry to $\Delta g_\parallel$ (Δg_z), resulting in a -229 ppt contribution (-224 from the main couplings). Each of Δg_x and Δg_y receive only one of these SO^2/SZ contributions, from $\hat{l}_y$ or $\hat{l}_x$, respectively, so the result is only -150 ppt for $\Delta g_\perp$ (-29 ppt originates from the weaker coupling with l_z operator transferred from $\Delta g_\parallel$ to $\Delta g_\perp$). As in the Ir d^7 complexes analyzed in the main text, the SO^2/SZ term contributes most to that component whose absolute value comes out smallest from SO/OZ; this is necessary due to the symmetry of the equations. Note, however, that in this case, the negative contribution to $\Delta g_\parallel$ brings it closer to $\Delta g_\perp$, not farther away. The SO^2/SZ term thus decreases the anisotropy of the g-tensor in the d^1 complex instead of enhancing it.

a)

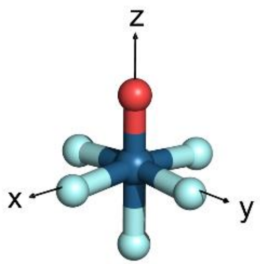

b)

	$\Delta g_{\perp}$	$\Delta g_{\parallel}$
total SO/OZ	**-417 ppt**	+70 ppt
total SO^2/SZ	-150 ppt	**-229 ppt**
total SO^2/OZ	+31 ppt	-81 ppt

c)

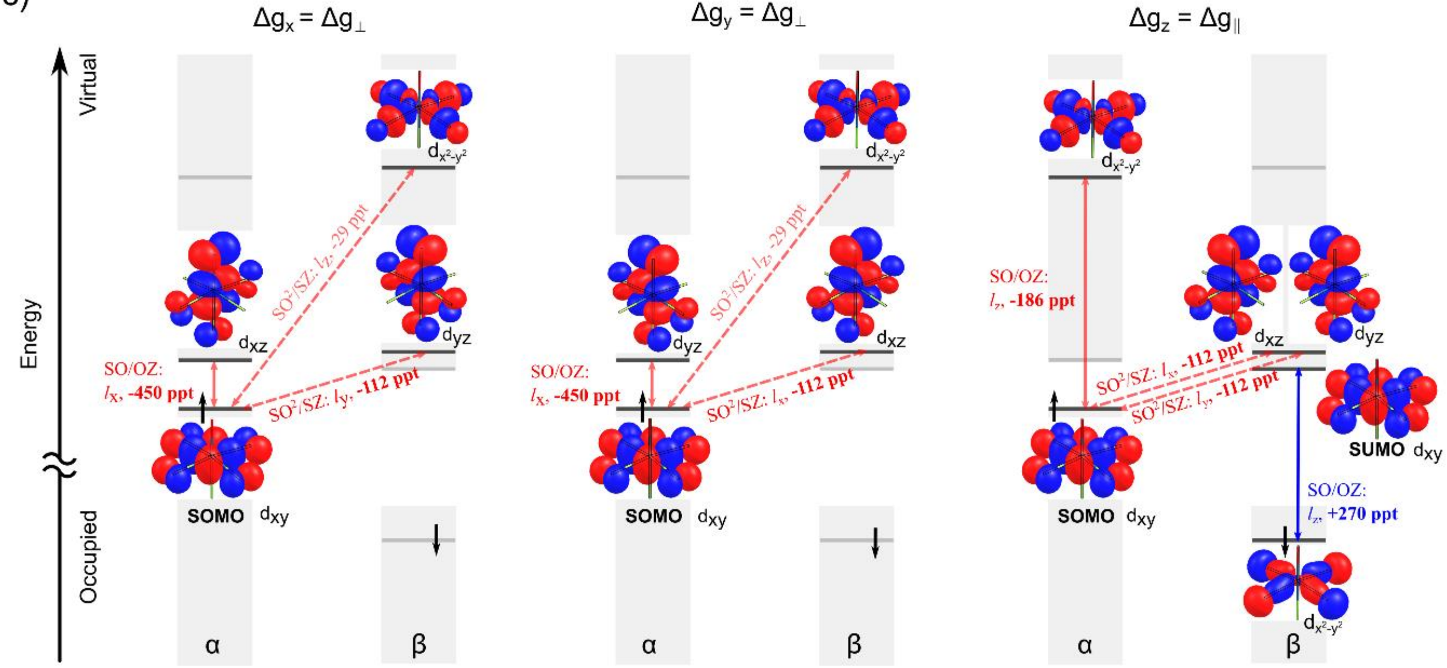

Figure S1. a) Orientation of the $OsOF_5$ molecule in the Cartesian axis system, b) summary of the individual contributions to the g-tensor calculated using PT/PBE/DZ/vacuum, and c) simplified molecular spin-orbital diagram of $OsOF_5$ from a nonrelativistic PBE/DZ/vacuum calculation with highlighted occupied↔vacant couplings which contribute at least 10 % of Δg_u^{term}. Individual g-shift components are analyzed separately. The optimized geometry of $OsOF_5$ was taken from a previous study by Gohr *et al.*[2] and used without further optimization.

g-tensor in d^5 Ir(IV) complex

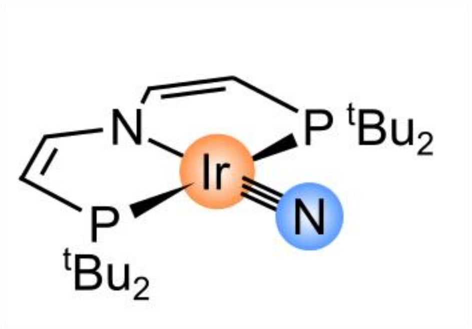

A slightly more complicated situation arises in the d^5 iridium(IV) complex. We only discuss it in general terms without analyzing the specific molecular spin-orbitals whose couplings contribute to the full values shown in Table S2 (an analysis of the MSO contributions to the SO/OZ g-shift was performed in a previous study by Bora *et al.*[6]). The SO/OZ term contributes mainly a large negative g-shift (-508 ppt) to Δg_x through an α↔α coupling. However, it also contributes a positive g-shift (+181 ppt) to Δg_y through a β↔β coupling. Both of these are transferred through the SO^2/SZ contribution to the perpendicular component Δg_z, *i.e.*, the middle component g_{22} ($g_{11} < g_{22} < g_{33}$), while the remaining two components are less affected. The anisotropy as measured by the span of the g-tensor (g_{33} - g_{11}) is thus not so clearly influenced by the quadratic term in this complex. It should be noted that the quadratic contributions are quite modest overall.

**Ir^{IV} ≡N**	Contribution	Δg_x	Δg_y	Δg_z
	SO/OZ	**-508**	**181**	66
	SO^2/OZ	-131	21	-9
	SO^2/SZ	-65	-94	**-158**
	Full PT	-704	108	-101

Table S3. Linear and quadratic SO contributions to individual components of the g-tensor obtained from analysis of the g-shifts (in ppt) for the Ir(IV) pincer compound[6] using the PT/PBE/DZ/vacuum approach.

Table S4 Experimental components of the g-shift (in ppt) and theoretical values calculated at the 4c level (TZ basis set) and using the PT approximation (DZ basis set).

			**Exp**			**4c/PBE0/TZ/vacuum**			**PT-DZ/vacuum**		
	Ligand (L)	Ref.	Δg_x	Δg_y	Δg_z	Δg_x	Δg_y	Δg_z	Δg_x	Δg_y	Δg_z
**1**	NH_2^-	7	120	730	-79	97	701	-122	62	698	-178
**2**	OH^-	8				135	907	-254	89	1095	-401
**3**	N_3^-	9	64	1089	-302	58	1002	-376	47	1090	-556
**4**	F^-	-				124	1033	-423	111	1516	-746
**5**	OH_2	-				-238	1220	-572	-490	1641	-1025
**6**	Cl^-	10[a]	-42	1348	-482	-134	1299	-641	-79	1695	-1053
**Rh6**	Cl^-	11	-179	1243	-318	-391	1067	-602	171	1024	-78
**7**	NH_3	7	-182	1358	-512	-399	1361	-745	-772	1936	-1451
**8**	Br^-	-				-202	1385	-719	-29	1671	-1188
**9**	CH_3^-	-				-551	1753	-977	-1132	3908	-2171
**Ir^{IV}**	≡N	9	-682	-117	-370	-496	-73	-286	-704	108	-101
**$OsOF_5$**		[b]	-387/ -318	-387/ -318	-197/ -185	-368	-368	-174	-536	-536	-239

[a] The principal components are not listed in Ref. [10]. [b] WF_6/Freon 133a

Effect of the implicit solvent model

Table S5. Experimental components of the g-shift (in ppt) and theoretical values calculated by using the 4c/PBE0/TZ/solvent (SOLV)[12] and 4c/PBE0/TZ/vacuum (VAC) approaches.

				EXP			SOLV			VAC	
	Ligand (*L*)	Ref.	solvent	Δg_x	Δg_y	Δg_z	Δg_x	Δg_y	Δg_z	Δg_x	Δg_y	Δg_z
**1**	NH_2^-	7	toluene	120	730	-79	124	669	-96	97	701	-122
**3**	N_3^-	9	toluene	64	1089	-302	53	1009	-382	58	1002	-376
**6**	Cl^-	10 [a]	Et_2O	-42	1348	-482	-76	1216	-574	-134	1299	-641
**7**	NH_3	7	THF	-182	1358	-512	-374	1389	-757	-399	1361	-745

[a] The simulated g-values were not listed in the paper,[10] but they can be estimated from the reported spectrum and were additionally confirmed by prof. Sven Schneider upon request.

Table S6. Contributions to the individual components of Δg (in ppt) up to the 3rd order: linear SO/OZ (OZ^1), quadratic SO^2/SZ (SZ^2), and SO^2/OZ (OZ^2) from the PT/PBE/DZ/vac calculation

		Δg_x				Δg_y				Δg_z			
	Ligand (L)	OZ^1	SZ^2	OZ^2	**PT**	OZ^1	SZ^2	OZ^2	**PT**	OZ^1	SZ^2	OZ^2	**PT**
**1**	NH_2	206	-300	157	**62**	836	-50	-87	**698**	214	-296	-96	**-178**
**2**	OH	344	-513	257	**89**	1341	-88	-158	**1095**	292	-525	-167	**-401**
**3**	N_3	378	-615	285	**47**	1374	-102	-182	**1090**	270	-639	-188	**-556**
**4**	F	521	-782	372	**111**	1923	-158	-249	**1516**	345	-847	-244	**-746**
**5**	OH_2	226	-1039	322	**-490**	2006	-79	-287	**1641**	280	-1028	-277	**-1025**
**6**	Cl	532	-1021	410	**-79**	2197	-181	-321	**1695**	336	-1098	-292	**-1053**
**7**	NH_3	222	-1396	402	**-772**	2399	-90	-373	**1936**	294	-1383	-362	**-1451**
**8**	Br	653	-1081	399	**-29**	2287	-262	-354	**1671**	325	-1239	-274	**-1188**
**9**	CH_3	506	-2091	453	**-1132**	4735	-155	-672	**3908**	365	-2129	-407	**-2171**
**Ir^{IV}**	≡N	-508	-65	-131	**-704**	181	-94	21	**108**	66	-158	-9	**-101**
**$OsOF_5$**		-417	-150	31	**-536**	-417	-150	31	**-536**	70	-229	-81	**-239**

Effect of the basis set and exact-exchange admixture

Table S7. Effect of the basis set on the components of Δg (in ppt) calculated by using the 4c/PBE0/xZ/vacuum approach.

		Δg_x			Δg_y			Δg_z		
	Ligand (*L*)	DZ	TZ	**EXP**	DZ	TZ	**EXP**	DZ	TZ	**EXP**
**1**	NH_2^-	73	97	**120**	745	701	**730**	-138	-122	**-79**
**2**	OH^-	94	135	**-**	978	907	**-**	-278	-254	**-**
**3**	N_3^-	-7	58	**64**	1097	1002	**1089**	-419	-376	**-302**
**4**	F^-	61	124	**-**	1137	1033	**-**	-460	-423	**-**
**5**	OH_2	-346	-238	**-**	1350	1220	**-**	-666	-572	**-**
**6**	Cl^-	-240	-134	**-42**	1438	1299	**1348**	-719	-641	**-482**
**7**	NH_3	-528	-399	**-182**	1504	1361	**1358**	-856	-745	**-512**
**8**	Br^-	-309	-202	**-**	1524	1385	**-**	-796	-719	**-**
**9**	CH_3^-	-644	-551	**-**	1886	1753	**-**	-1048	-977	**-**

Table S8. Effect of the exact-exchange admixture in the PBE functional on the components of Δg (in ppt) calculated by using the 4c/PBEx/DZ/vacuum approach.

		Δg_x			Δg_y			Δg_z		
	Ligand (*L*)	PBE	PBE0	**EXP**	PBE	PBE0	**EXP**	PBE	PBE0	**EXP**
**1**	NH_2^-	77	73	**120**	559	745	**730**	-86	-138	**-79**
**2**	OH^-	127	94	**-**	709	978	**-**	-175	-278	**-**
**3**	N_3^-	88	-7	**64**	757	1097	**1089**	-262	-419	**-302**
**4**	F^-	170	61	**-**	777	1137	**-**	-306	-460	**-**
**5**	OH_2	-151	-346	**-**	933	1350	**-**	-395	-666	**-**
**6**	Cl^-	12	-240	**-42**	961	1438	**1348**	-449	-719	**-482**
**7**	NH_3	-225	-528	**-182**	1013	1504	**1358**	-484	-856	**-512**
**8**	Br^-	21	-309	**-**	979	1524	**-**	-495	-796	**-**
**9**	CH_3^-	-290	-644	**-**	1345	1886	**-**	-658	-1048	**-**

REFERENCES

(1) Repisky, M.; Komorovsky, S.; Malkin, E.; Malkina, O. L.; Malkin, V. G. Relativistic Four-Component Calculations of Electronic g-Tensors in the Matrix Dirac–Kohn–Sham Framework. *Chem. Phys. Lett.* **2010**, *488* (1), 94–97.

(2) Gohr, S.; Hrobarik, P.; Repisky, M.; Komorovsky, S.; Ruud, K.; Kaupp, M. Four-Component Relativistic DFT Calculations of EPR g- and Hyperfine-Coupling Tensors Using Hybrid Functionals: Validation on Transition-Metal Complexes with Large Tensor Anisotropies and Higher-Order Spin-Orbit Effects. *J. Phys. Chem. A* **2015**, *119* (51), 12892–12905. https://doi.org/10.1021/acs.jpca.5b10996.

(3) Komorovsky, S. *Relativistic Effects and the Chemistry of Heavy Elements*, submitted.; Elsevier, 2022.

(4) Mabbs, F. E.; Collison, D. *Electron Paramagnetic Resonance of d Transition Metal Compounds*, 1st edition.; Elsevier Science: Amsterdam, 2013.

(5) Holloway, J. H.; Hope, E. G.; Raynor, J. B.; Townson, P. T. Magnetic Resonance Studies on Osmium Pentafluoride Oxide. *J. Chem. Soc., Dalton Trans.* **1992**, No. 7, 1131. https://doi.org/10.1039/dt9920001131.

(6) Bora, P. L.; Novotný, J.; Ruud, K.; Komorovsky, S.; Marek, R. Electron-Spin Structure and Metal–Ligand Bonding in Open-Shell Systems from Relativistic EPR and NMR: A Case Study of Square-Planar Iridium Catalysts. *J. Chem. Theory Comput.* **2019**, *15* (1), 201–214. https://doi.org/10.1021/acs.jctc.8b00914.

(7) Scheibel, M. G.; Abbenseth, J.; Kinauer, M.; Heinemann, F. W.; Würtele, C.; de Bruin, B.; Schneider, S. Homolytic N–H Activation of Ammonia: Hydrogen Transfer of Parent Iridium Ammine, Amide, Imide, and Nitride Species. *Inorg. Chem.* **2015**, *54* (19), 9290–9302. https://doi.org/10.1021/acs.inorgchem.5b00829.

(8) Delony, D.; Kinauer, M.; Diefenbach, M.; Demeshko, S.; Würtele, C.; Holthausen, M. C.; Schneider, S. A Terminal Iridium Oxo Complex with a Triplet Ground State. *Angewandte Chemie International Edition* **2019**, *58* (32), 10971–10974. https://doi.org/10.1002/anie.201905325.

(9) Scheibel, M. G.; Askevold, B.; Heinemann, F. W.; Reijerse, E. J.; Bruin, B. de; Schneider, S. Closed-Shell and Open-Shell Square-Planar Iridium Nitrido Complexes. *Nat. Chem.* **2012**, *4* (7), 552–558. https://doi.org/10.1038/nchem.1368.

(10) Meiners, J.; Scheibel, M. G.; Lemée-Cailleau, M.-H.; Mason, S. A.; Boeddinghaus, M. B.; Fässler, T. F.; Herdtweck, E.; Khusniyarov, M. M.; Schneider, S. Square-Planar Iridium(II) and Iridium(III) Amido Complexes Stabilized by a PNP Pincer Ligand. *Angew. Chem. Int. Ed.* **2011**, *50* (35), 8184–8187. https://doi.org/10.1002/anie.201102795.

(11) Scheibel, M. G.; Wu, Y.; Stückl, A. C.; Krause, L.; Carl, E.; Stalke, D.; de Bruin, B.; Schneider, S. Synthesis and Reactivity of a Transient, Terminal Nitrido Complex of Rhodium. *J. Am. Chem. Soc.* **2013**, *135* (47), 17719–17722. https://doi.org/10.1021/ja409764j.

(12) Remigio, R. D.; Repisky, M.; Komorovsky, S.; Hrobarik, P.; Frediani, L.; Ruud, K. Four-Component Relativistic Density Functional Theory with the Polarisable Continuum Model: Application to EPR Parameters and Paramagnetic NMR Shifts, 2016. https://doi.org/10.6084/m9.figshare.4039764.v1.
